# Mechanical Valve Thrombosis Secondary to Severe Acute Respiratory Syndrome Coronavirus 2 Infection: A Case Report

**DOI:** 10.7759/cureus.23358

**Published:** 2022-03-21

**Authors:** Carolina Cardona Buitrago, Aida Maired Builes Gutierrez, David Jiménez Marín, Camilo Aristizábal García

**Affiliations:** 1 Internal Medicine, Hospital San Vicente Fundación, Rionegro, COL; 2 Epidemiology, Hospital San Vicente Fundación, Rionegro, COL; 3 General Medicine, Unidad Médica Integral (UMI), Marinilla, COL

**Keywords:** sars-cov-2, covid-19, thrombosis, mitral valve, coronavirus infections

## Abstract

Although the association of coronavirus disease 2019 (COVID-19) and thromboembolic disease is well known, cases of severe acute respiratory syndrome coronavirus 2 (SARS-CoV-2) infection and mechanical valve thrombosis have not been described enough. Mechanical valve thrombosis is a medical emergency that is associated with a great impact on patients' morbidity and mortality. Here, we report a case of a patient with mechanical valve thrombosis secondary to SARS-CoV-2 infections that required valve replacement with satisfactory postoperative recovery.

A 52-year old female patient was presented with a previously implanted mechanical prosthesis (type - St. Jude Medical 29 mm; St. Paul, MN: St. Jude Medical, Inc.) eight years ago due to rheumatic fever, under anticoagulation with warfarin and valvular atrial fibrillation (permanent), congenital single kidney (glomerular filtration rate {GFR}: 89.9 mL/min), and hypothyroidism. She was admitted to the hospital with a high level of complexity due to respiratory difficulty and generalized edematous syndrome, and a reverse transcription-polymerase chain reaction (RT-PCR) confirmed COVID-19 infection (20 days before admission); the patient was anticoagulated with warfarin (international normalized ratio {INR} at admission was 2.63 seconds). As per protocol, a CT-chest scan tomography was performed and showed organized pneumonia in the right apical lobe. We performed a transesophageal echocardiogram, which showed a thrombus (20 x 15 x 20 mm) in the lateral disc of the mechanical prosthesis, restricting its mobility. The patient presented signs of hypoperfusion (lactate levels: 4 mmol/L; urine per hour: 1 cc/kg) with associated low cardiac output syndrome, requiring double vasopressor support at the maximum dose (achieving a mean arterial pressure of 72 mmHg) due to the clinical condition and the large size of the thrombus, the cardiovascular surgeon, in agreement with the family, decides to carry out emergency valve replacement surgery with replacement of a mechanical prosthesis replacement (St. Jude No. 29; St. Paul, MN: St. Jude Medical, Inc.). The patient presented a satisfactory postoperative recovery, achieving INR goals, with subsequent discharge and follow-up at two months with transthoracic ultrasound, where normofunctional mitral prosthesis was demonstrated, without evidence of thrombi or intracavitary masses.

Mechanical mitral valve thrombosis, secondary to SARS-CoV-2 infection is a serious complication with poor prognosis that requires a high rate of suspicion, and timely diagnostic aids are essential to confirm the diagnosis. Managing this issue should be interdisciplinary and individualized considering the clinical condition of the patient and the associated comorbidities.

## Introduction

Severe acute respiratory syndrome coronavirus 2 (SARS-CoV-2) infection poses a huge challenge to the world both because of the morbidity and mortality presented in the context of acute respiratory distress syndrome and because of the presence of associated epiphenomena, among these, thrombotic complications are well-described in literature, especially in the venous circulation, up to 25-43% in ICU patients [1-3].

There are also reports of arterial thrombosis, including in the central nervous system (CNS). The largest study, which included 3334 individuals reported stroke in 1.6% and myocardial infarction in 8.9% [4]. Arterial thrombotic events were associated with increased mortality (adjusted HR: 1.99; 95% CI: 1.65-2.40), limp ischemia, and microvascular thrombosis are also described, and finally, there is an increase in hemostatic disorders such as bleeding that occurs with less prevalence than the previous ones [4].

Here, we describe a rare case of mechanical valve thrombosis in the era of coronavirus disease 2019 (COVID-19) pandemic, which carries a great impact on length of stay and overall cost, besides the increased morbidity and mortality. We highlight the lack of solid evidence regarding this case scenario.

## Case presentation

**Figure 1:**
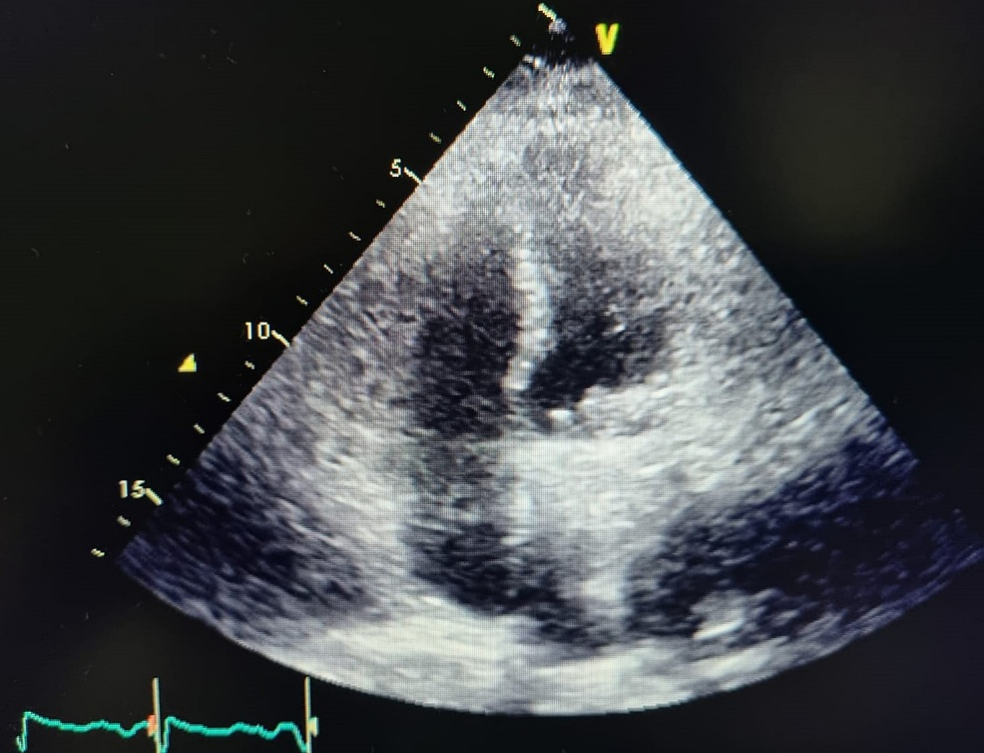
Color Doppler echocardiogram A hyperechogenic (with acoustic shadowing), mobile image of 7 x 9 mm is shown at the lateral level of the mitral prosthesis toward the ventricular side, one of the discs showed excursion without excursion of the second disc.

A 52-year-old female patient was admitted to the hospital with a high-complexity diagnosis of COVID-19 demonstrated by polymerase chain reaction (PCR) taken at another institution. On admission, the patient had severe hypoxemia (SO_2_ 48% per room air) and was given supplemental 50% oxygen via a Venturi mask; also, she had a generalized edematous syndrome. The patient had a personal history of rheumatic fever; a mechanical prosthetic mitral valve replacement was done eight years ago (type - St. Jude Medical 29 mm; St. Paul, MN: St. Jude Medical, Inc.), the patient was anticoagulated with warfarin (international normalized ratio {INR} at admission was 2.63 seconds) and had valvular permanent atrial fibrillation, obesity, congenital single kidney, and hypothyroidism. Because the patient was admitted with acute dyspnea and desaturation, it was decided to perform simple non-contrast chest CT with evidence of a pattern of organizational pneumonia of right apical predominance, non-invasive mechanical ventilation (NMIV), and administration of parenteral steroid was initiated, presenting clinical improvement and significant oxygenation. Therefore, it is defined to suspend NMIV and leave with low flow device; also, pulmonary embolism was ruled out due to computed tomography angiography (CTA). Due to the clinical evolution of the patient, it was decided to continue her treatment with IV furosemide and a transthoracic echocardiogram was requested because the patient persisted with congestive symptoms. A hyperechogenic, mobile image of 7 x 9 mm was shown at the lateral level of the mitral prosthesis toward the ventricular side, one of the discs showed excursion without excursion of the second disc. Transesophageal echocardiogram was performed to better characterize the findings, where mechanical double-disc prostheses were shown dysfunctional in the mitral position by thrombus in the lateral disk that prevented its mobility, with thrombus of 20 x 15 mm x 20 mm with the maximum gradient of 39, mean gradient of 22 mmHg, effective hole area estimated by pressure half time (THP) of 1.2 cm^2^ (Figure 1).

**Figure 2:**
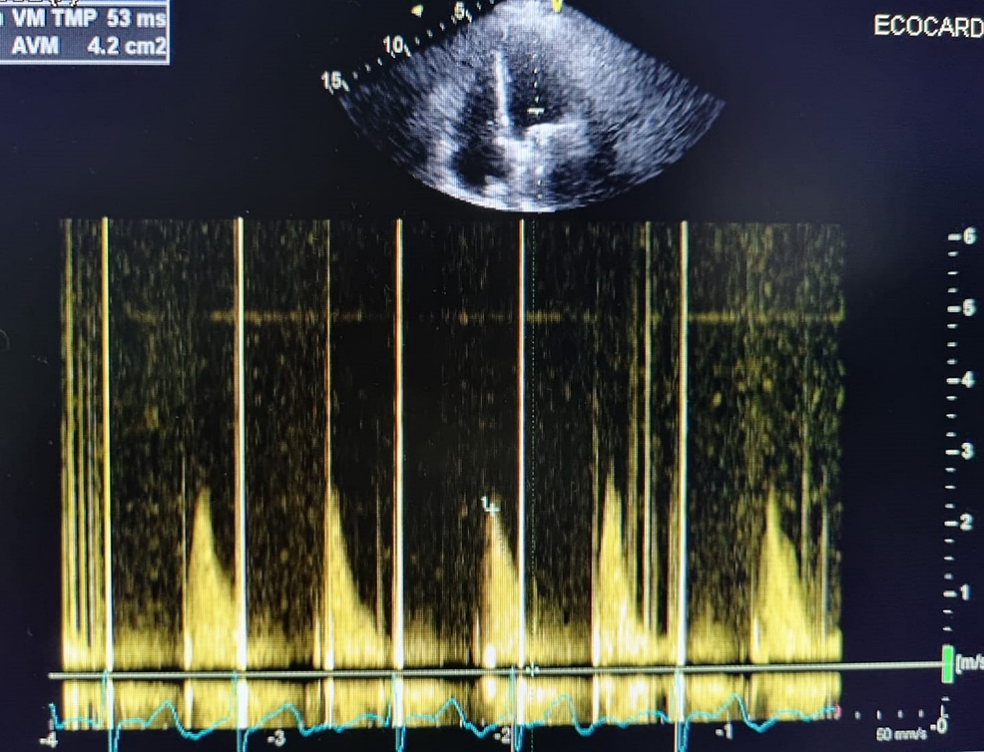
Color Doppler echocardiogram After two months, a transthoracic ultrasound showed that normofunctional mitral prosthesis is found with no evidence of thrombi or intracavitary masses.

The patient presented signs of hypoperfusion with associated low output syndrome, requiring double vasopressor support at the maximum dose, due to the clinical condition and the large size of the thrombus; the cardiovascular surgeon, in agreement with the family, decides to carry out emergency valve replacement surgery with replacement of a mechanical prosthesis replacement (St. Jude No. 29 {St. Paul, MN: St. Jude Medical, Inc.}, extracorporeal circulation {ECC} time 140 min, Clamp time 114 min femoral artery reconstruction with anastomosis). The patient presented a satisfactory postoperative recovery and was transferred to the ICU for monitoring and care. During her stay in the ICU, she presented significant bradyarrhythmia with rhythm of escape from the junction that resolved spontaneously and without hemodynamic compromise. Holter was therefore performed where occasional premature ventricular complexes (PVCs) of two morphologies, pairs, bigeminy, and episodes of non-sustained ventricular tachycardia (NSVT) were found as abnormal findings, hence, it was necessary as an integral part of her treatment to initiate beta-blocker and antiarrhythmics. In addition, due to the findings of the Holter, it was decided to implant unicameral pacemaker by electrophysiology. The clinical course was favorable, and the patient was discharged when he reached the therapeutic INR (INR at hospital discharge was 3.57 seconds). Two months later, a follow-up transthoracic echocardiogram was performed which showed that normofunctional mitral prosthesis is found (mechanical prosthesis in mitral position with adequate mobility of its hemidiscos, adequate transprosthetic gradients) with no evidence of thrombi or intracavitary masses (Figure 2).

## Discussion

Since the beginning of the COVID-19 pandemic, several research articles have been published showing an association between severe COVID and hypercoagulability. The most common coagulopathy pattern found is characterized by elevated levels of fibrinogen D-dimer [5,6]. Coagulopathy appears to be related to disease severity and associated inflammation, and not to intrinsic viral activity [5,7]. It is suggested that COVID-19 infection triggers an excessive activation of the immune system, causing the release of different inflammatory mediators, among them are cytokines, which interact with platelets and different coagulation proteins, promote thrombogenesis and cause damage to the microvascular system [5].

The other possible factors of COVID-19-associated thrombosis include hypoxia, prolonged hospitalization in the ICU, age, and obesity. Although the exact mechanism of thrombus formation is not clear in this case, predisposing factors are found, such as a history of paroxysmal valvular atrial fibrillation (incidence of thrombus formation of 10-15%), obesity, and COVID-19 infection.

The prevalence of venous thromboembolic disease varies in different published studies, but it is estimated between 25% and 54% conditioned by methodological differences, the use of thromboprophylaxis and the test used for diagnosis [5,8-10]. On the other hand, prosthetic valve thrombosis is currently rare, given the strict control of oral anticoagulation in these patients [11]. The annual rate of thrombosis in mechanical prostheses in mitral position is 5/1000 patients/year [11].

The association between SARS-CoV-2 infection and mechanical valve thrombosis has been slightly described. In the case reported by Gisbert et al., a 77-year-old male patient with biological mitral prosthetic valve thrombosis secondary to COVID-19 was treated adequately with standard treatment for heart failure with diuretics and anticoagulation for 15 days with low molecular weight heparins [12].

Steven et al. published a case of thrombosis of aortic bioprosthesis in a 79-year-old woman with COVID-19; treatment was performed with an infusion of unfractionated heparin and then warfarin. A three-month follow-up was described with transthoracic ultrasound with a return to baseline aortic valve gradient [13].

Vinnakota et al. reported a case of a 63-year-old female patient, in which a thrombus was detected within the mitral bioprosthesis, with almost total occlusion of the inflow, with refractory cardiogenic shock and with prohibitive cardiac risk. Intravenous tenecteplase was urgently administered with almost complete resolution of 90 minutes after thrombolysis [14].

Prosthetic thrombosis may be non-obstructive or obstructive. In the case of non-obstructive thrombosis, its main form of presentation is with hemodynamic compromise, ranging from progressive dyspnea to cardiogenic shock [11]. This form of thrombosis was presented by Vinnakota et al. and by the patient in this study. As a hypothesis, it is proposed that thrombotic phenomena associated with COVID-19 can occur after the acute phase of infection as in the case described by Gisbert et al., and these can occur despite the administration of anticoagulant treatment, both situations present in this case [12].

As for the treatment of prosthetic valve thrombosis, it constitutes an emergency that can be treated surgically through replacement of the affected valve, or through systematic fibrinolytic therapy. Both procedures are endorsed by current guidelines from the American Heart Association/American College of Cardiology (ACC/AHA) as amended in 2017 [15,16]. However, guidelines from the European Society of Cardiology and the European Association of Cardio-Thoracic Surgery (ESC/EACTS) recommend surgery in severe cases without serious comorbidity (class I, level C) [15].

## Conclusions

In conclusion, it is crucial not to oversee the dyspnea as a lone COVID-19 infection symptom in complex patients, such as patients with valve prosthesis. A high level of suspicion allows a timely diagnosis and treatment that must be individualized considering the findings, the feasibility of fibrinolysis, and the risk of eventual surgery. And, it is in this scenario where echocardiography has a fundamental role to assess the status of the valves as well as to determine alterations associated with them, determine myocardial functionality and indirect signs of pulmonary thromboembolism, which is why we suggest performing this test in patients with COVID-19 and valve prosthesis.
